# Scientific Artisan

**Published:** 1859-11

**Authors:** 


### SCIENTIFIC ARTISAN.

This weekly publication, issued by the American Patent Company, is one highly valuable to all inventors, tradesmen, artists, and mechanics of all kinds ; as all subjects pertaining to mechanics are freely and very thoroughly discussed in the full light of modern science ; every subject discussed in it bears evidence of a thorough acquaintance with scientific principles. It is a publication that no mechanic or artist should be without.

This Company affords unusual facilities to inventors for the procurement of patents. Address Scientific Artisan, or American Patent Company, Cincinnati, O. T.

